# Design of a Portable Nondestructive Instrument for Apple Watercore Grade Classification Based on 1DQCNN and Vis/NIR Spectroscopy

**DOI:** 10.3390/mi16121357

**Published:** 2025-11-29

**Authors:** Haijian Wu, Yong Lin, Wenbin Zhang, Zikang Cao, Chunlin Zhao, Zhipeng Yin, Yue Lu, Liju Liu, Ding Hu

**Affiliations:** 1 School of Mechanical and Electrical Engineering, Kunming University of Science and Technology, Kunming 650500, China; wuhaijian@stu.kust.edu.cn (H.W.);; 2 Department of Scientific Research, Zhijiang College of Zhejiang University of Technology, Shaoxing 312030, China; 3 School of Mechanical and Electrical Engineering, Kunming University, Kunming 650214, China; 4 College of Power and Energy Engineering, Harbin Engineering University, Harbin 150001, China; 5 College of Mechanical and Electrical Engineering, Xi’an Jiaotong University, Xi’an 710049, China

**Keywords:** portable, online nondestructive detection, apple watercore, visible/near-infrared spectroscopy, 1DQCNN

## Abstract

To address the challenge of nondestructively identifying watercore disease in apples during growth and maturation, a portable device was developed for real-time grading of apple watercore using visible/near-infrared (Vis/NIR) spectroscopy combined with a one-dimensional quadratic convolutional neural network (1DQCNN). The instrument enables rapid, nondestructive, and accurate detection of apple watercore grades. The AI-OX2000-13 micro-spectrometer is used as the core data acquisition unit, and an ARM processing system is built with the STM32F103VET6 as the main control chip. A 4G wireless communication module enables efficient and stable data transmission between the processor and computer, meeting the real-time detection needs of apple watercore content in orchard environments. To improve the scientific and accurate classification of watercore grades, this paper combines the BiSeNet and RIFE algorithms to construct a 3D model of apple watercore, allowing quantification of the degree of watercore and classification into four levels. Based on this, quadratic convolution operations are incorporated into a one-dimensional convolutional neural network (1DCNN), leading to the development of the 1D quadratic convolutional neural network (1DQCNN) model for watercore grade classification. Experimental results indicate that the model achieves a classification accuracy of 98.05%, outperforming traditional methods and conventional CNN models. The designed portable instrument demonstrates excellent accuracy and practicality in real-world applications.

## 1. Introduction

The apple industry plays a significant role in the agricultural economy [1]. It is a major fruit in many countries worldwide, and in recent years, apple production has rapidly increased in Asia and Africa [2,3]. Apples are abundant in water, sugars, vitamins, minerals, and antioxidants, which contribute to their high nutritional value and support human health, including the enhancement of immune function [4]. The quality of apples is influenced by various factors [5,6], and one of the key factors is apple watercore, also known as “sugar core disease” or “honey apple disease” [7]. Watercore apples, as a type of high-quality apple, are characterized by their unique taste and limited production, making them highly favored by consumers [8]. Watercore primarily develops due to pronounced diurnal temperature differences in high-altitude regions, which hinder the timely conversion of sorbitol transported from the leaves, resulting in its accumulation around the fruit core and vascular bundles [9]. A key feature of Watercore is the appearance of translucent, watery substances between the flesh cells near the core and vascular bundles during the fruit’s maturation stage [10]. Watercore in apples is undetectable during the growth period, but Watercore apples have higher product value than normal apples. Therefore, accurately detecting the Watercore content in apples during their growth and maturation is crucial for guiding the harvesting process, improving the individual value of apples, and ultimately increasing the income of apple growers.

At present, apple watercore detection mainly relies on manual cutting or visual inspection [11], both of which are either destructive or inaccurate. To achieve nondestructive and accurate detection, methods such as thermal imaging, CT, and MRI have been explored [12,13], but they are limited by long detection times, high cost, and low accuracy [14]. In contrast, Vis/NIR spectroscopy offers a more balanced solution in terms of spectral information acquisition, instrument portability, and real-time detection. Although fluorescence techniques have advantages in detecting specific compounds, their sensitivity to excitation light and environmental conditions limits their practicality for on-site apple watercore detection. With the development of visible/near-infrared (Vis/NIR) spectroscopy [15], this technique has been widely applied for nondestructive internal quality assessment of fruits and vegetables due to its efficiency, noninvasiveness, and ease of operation [16,17,18,19,20]. Guo Zhimin et al. [21] combined NIR spectroscopy and imaging with multiple algorithms to evaluate sugar content and moisture. Khodabakhshian R et al. [22] applied Vis/NIR with multivariate regression to predict pomegranate maturity and quality. Mancini M et al. [23] used Vis/NIR and PLSR to determine strawberry sugar content and firmness, while Egei M et al. [24] predicted tomato soluble solids and lycopene. Ghooshkhaneh N G et al. [25] integrated NIR and SVM for early detection of citrus heart rot, demonstrating the potential of Vis/NIR for disease and quality assessment. These studies confirm that Vis/NIR spectroscopy is an effective tool for rapid, nondestructive fruit evaluation, and thus this study adopts it to detect and classify apple watercore levels.

Of course, this technology has also achieved substantial research progress in the detection of apples. Tian X et al. [26] analyzed spectral data of moldy-core and healthy apples within the wavelength range of 500–1000 nm. Using principal component analysis (PCA) and partial least squares regression (PLSR) models, they effectively distinguished moldy-core apples from healthy ones, achieving a detection accuracy exceeding 92%. Huang Hua et al. [27] investigated a prediction method for soluble solid content (SSC) in apples at different maturity stages based on Vis/NIR spectroscopy and a functional linear regression model. Using spectral data from 400–1000 nm, the analysis achieved a prediction accuracy above 97.5%. Zhao C et al. [28] improved the accuracy of apple watercore detection by integrating visible/near-infrared (Vis/NIR) spectroscopy with Gramian Angular Field (GAF) encoding and the ConvNeXt deep learning model. Chang H et al. [29] proposed an online evaluation method for apple watercore based on Vis/NIR spectroscopy. Using spectral data in the 400–1000 nm range and analyzing it with a PLSR model, they effectively distinguished apples with and without watercore, reaching a detection accuracy of 92.5%. Zhang et al. [30] developed a watercore identification method for Xinjiang Ice-Sugar Red Fuji apples using manifold learning and near-infrared transmittance spectroscopy. The approach extracted characteristic information of apple watercore through manifold learning algorithms, effectively differentiating healthy apples from those affected by watercore. Ma Y et al. [31] proposed an OCRNet-based approach for detecting apple watercore, targeting apples cultivated in the cold and cool regions of Yunnan Province. Their research employed deep learning algorithms to enhance the accuracy of watercore detection. The aforementioned studies have utilized visible/near-infrared (Vis/NIR) spectroscopy in combination with advanced mathematical modeling techniques to achieve nondestructive evaluation of apple quality. However, most of these investigations were carried out under controlled laboratory environments with single-factor analyses. Quantitative characterization methods for complex structural defects such as watercore remain underdeveloped, and the existing models still require further enhancement in data processing capacity and detection precision.

With the remarkable enhancement of computing power and the rapid evolution of computer vision technologies, artificial intelligence approaches—particularly deep learning—have achieved significant breakthroughs in intelligent agricultural detection. Deep neural networks based on end-to-end data-driven architectures have found extensive applications. As a core framework of deep learning, the Convolutional Neural Network (CNN), initially introduced by Lecun et al. in 1998 [32], has evolved into several representative models such as AlexNet [33], VGGNet [34], ResNet [35], and Mingxing Tant [36]. These networks have demonstrated exceptional performance in fields including image recognition, speech analysis, and spectral data interpretation. However, most existing deep learning methods still rely on two-dimensional spectral images or manually extracted features, which limits their ability to capture the sequential and nonlinear properties of spectral data and constrains model stability and generalization. Compared with ConvNeXt and other CNN-based architectures, the key innovation of 1DQCNN lies in the use of quadratic convolutions to directly extract higher-order features from one-dimensional spectral data. This design not only reduces data dimensionality but also preserves spectral integrity while enhancing the model’s representational power and feature extraction capability, thereby enabling more accurate and efficient classification of apple watercore grades.

To overcome these limitations, this study develops a one-dimensional quadratic convolutional neural network (1DQCNN) by incorporating quadratic convolution operations into the conventional one-dimensional convolutional neural network (1D-CNN) framework. Using one-dimensional visible/near-infrared (Vis/NIR) spectral data as input, the proposed method eliminates the need for spectral two-dimensionalization, thereby enhancing modeling efficiency and demonstrating improved noise tolerance, stability, and generalization performance. To provide the 1DQCNN model with a scientific and accurate basis for watercore level classification, this study introduces an image reconstruction-based method for quantifying the degree of watercore. This method reconstructs the three-dimensional internal structure of apple watercore through image recognition and slice-stacking algorithms and calculates the ratio of the watercore region to the total fruit volume, thereby achieving a scientifically grounded classification of apple watercore levels. Consequently, the method provides more precise and reliable classification labels for the deep learning model, substantially enhancing the detection accuracy and practical value of the 1DQCNN model.

In addition, most of the above studies are limited to laboratory environments, where the detection devices are typically large and complex, making them unsuitable for rapid on-site detection, and research on portable detection devices remains relatively weak. To overcome this limitation, recent research has increasingly focused on developing portable detection systems utilizing visible/near-infrared (Vis/NIR) spectroscopy. Guo Ya et al. [37] developed a portable nondestructive apple sweetness detector using near-infrared spectroscopy. Operating in the 800–1100 nm spectral range and utilizing a partial least squares regression (PLSR) model for sugar content prediction, the device achieves rapid and nondestructive assessment of apple sweetness with an accuracy above 93%. The device is compact and easy to operate, suitable for on-site rapid detection. Fan S et al. [38] developed a portable Vis/NIR device for nondestructive evaluation of apple soluble solid content (SSC). The device utilizes Vis/NIR spectroscopy combined with a PLSR model to predict SSC, offering good accuracy and stability, while being lightweight and easy to operate for on-site rapid detection. Guo Z et al. [39] independently developed a portable visible/near-infrared (Vis/NIR) transmission system combined with chemometric techniques for nondestructive evaluation of apple quality attributes, including soluble solids content (SSC), watercore level, firmness, and pH. By preprocessing spectral data and combining it with a CARS-CNN model, the study improved prediction accuracy, providing a convenient and efficient nondestructive method with strong potential for field application. Although these studies leverage the nondestructive, efficient, and environmentally friendly advantages of Vis/NIR spectroscopy to achieve nondestructive detection of internal indicators such as apple sweetness, SSC, and watercore content, several limitations remain: first, the detection devices are still relatively large and inconvenient to operate, limiting rapid detection and promotion in large orchards; second, apple detection generally still requires harvesting the fruit, preventing real-time, in situ measurements on trees; third, the prediction models require further improvement in convenience and accuracy for analyzing apple watercore data. Therefore, developing a portable, efficient, accurate, and field-deployable online apple watercore detection device is of great significance, as it would enhance orchard management, improve harvesting and sorting efficiency, enable apple grading and price optimization, and promote the apple industry toward precise, high-quality production.

**Figure 1:**
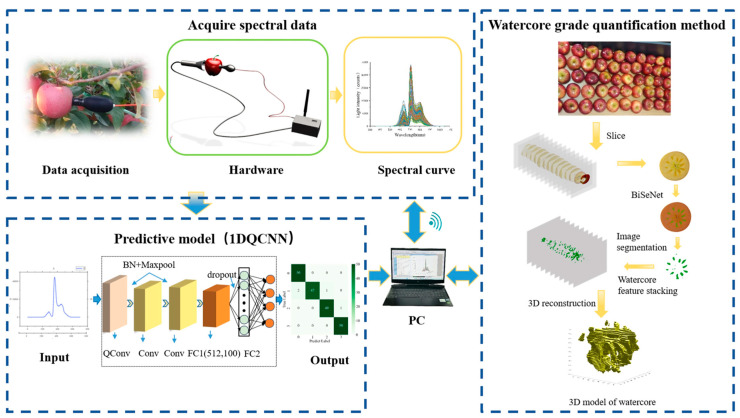
Schematic Diagram of the Portable Online Nondestructive Apple Watercore Detection Instrument.

Based on the above analysis, this study designed and developed a portable on-line nondestructive detection device for apple watercore grading by integrating a one-dimensional quadratic convolutional neural network (1DQCNN), visible/near-infrared (Vis/NIR) spectroscopy, and microcontroller technology: (1) Nondestructive and portable: the device uses Vis/NIR spectroscopy to achieve nondestructive detection of apple watercore, with a compact housing size of only 147 mm × 91 mm × 63 mm and simple operation; (2) High efficiency and accuracy: the device establishes a stable and efficient connection between the ARM-based core processor and a computer via a 4G wireless module, and combines the 1DQCNN model with a novel apple watercore quantification method to accurately detect and classify apple watercore levels; (3) On-line field detection: the device enables real-time, in situ detection and analysis of apple watercore directly on the tree. A schematic diagram of the proposed research method is shown in Figure 1.

## 2. Instrument Design

### 2.1. Working Principle

**Figure 2:**
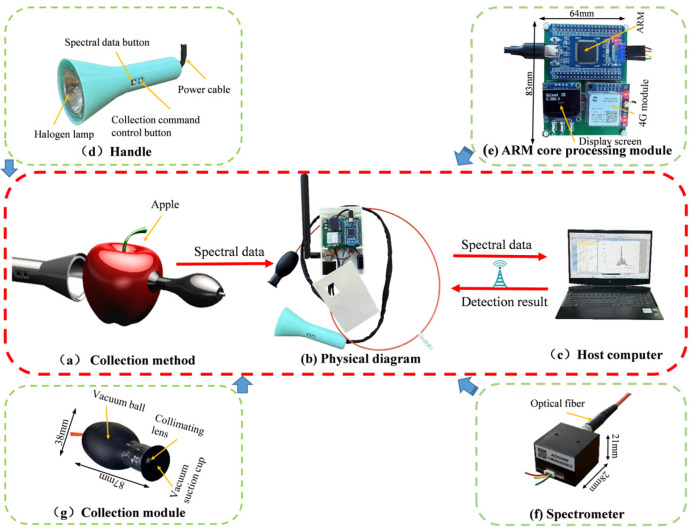
Working Principle of the Portable Online Nondestructive Apple Watercore Grading Device.

The working principle of the portable apple watercore nondestructive detection instrument is shown in Figure 2. During the spectral data acquisition, the vacuum suction cup (Figure 2g) is attached to the equatorial surface of the apple sample. The halogen light source (Figure 2d) is positioned on the opposite side of the spectral collection probe, also illuminating the apple sample along the equatorial plane. The transmitted light passes through the apple’s flesh, carrying the spectral information of its internal structure, and is received by the spectral collection probe. After passing through the apple flesh, the light is collected by the spectral probe and transmitted via optical fiber to the spectrometer (Figure 2f). The spectral signal collected by the spectrometer is converted into a digital signal by the ARM processor (Figure 2e) and sent to the 4G module. Subsequently, the 4G module transmits the digital signal to the host computer for storage. The host computer uses the prediction model based on the one-dimensional quadratic convolutional neural network (1DQCNN) to determine the Watercore level of the apple sample. Finally, the prediction result is sent back through the 4G module to the ARM processor, and the result is displayed in real time on the screen.

### 2.2. Hardware Design

**Figure 3:**
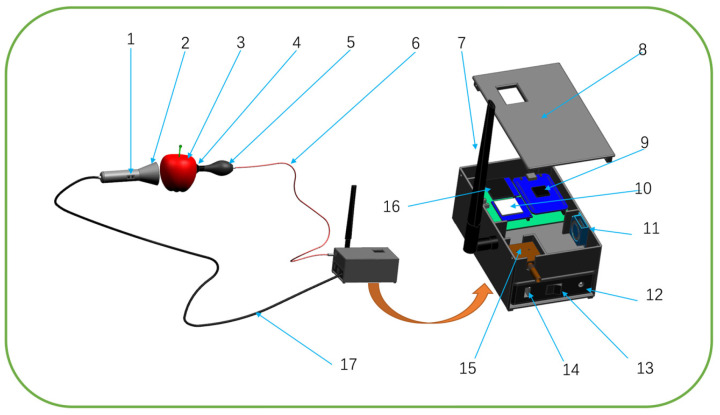
Structural Diagram of the Portable Nondestructive Apple Watercore Detection Device. 1. Control Button; 2. Halogen Lamp; 3. Apple; 4. Vacuum Suction Cup; 5. Vacuum Bulb; 6. Optical Fiber; 7. Antenna; 8. Enclosure; 9. ARM Processor; 10. Fourth-generation Module; 11. Heat Sink; 12. 5 V Port; 13. Power Switch; 14. 12 V Port; 15. Spectrometer; 16. Display Screen; 17. Power Cable.

To meet usage requirements, the shell and handle were manufactured using 3D printing. Figure 2b shows the physical image of the portable detection device. The overall housing dimensions of the instrument are 150 × 90 × 63 mm, and the total weight of the device is 853 g. The antenna position can be adjusted flexibly according to the user’s needs, and the optical fiber length is 1.5 m. The hardware system of the instrument mainly consists of the following components: spectrometer (AIOX2000-13, Yuguang Technology Co., Ltd., Beijing, China), ARM core control board (including STM32 core processing board, 4G communication module, and display screen), computer, light source system (halogen lamp), light collection system (including vacuum sphere, vacuum suction cup, and collimating mirror), power system (12 V and 5 V DC power supply), and other external circuits. A 3D diagram of the hardware is shown in Figure 3.

#### 2.2.1. The Design of ARM Processing Module

The module primarily comprises an STM32F103VET6 microcontroller (Julichuang Information Technology Co., Ltd., Xi’an, China), a 0.96-inch OLED display, and a Silverda Air724 wireless transmission unit (Silverda Electronics Co., Ltd., Shenzhen, China). The STM32F103VET6 integrates a high-performance 32-bit ARM Cortex-M3 core operating at up to 72 MHz, providing strong computational capability. It includes 512 KB of Flash memory, 64 KB of SRAM, and a UART peripheral interface. The module’s main function is to convert the analog signals from the spectrometer into digital form and transmit them to the 4G communication unit via the UART interface. The 4G module then uploads the data received from the STM32F103VET6 to the host computer and simultaneously receives the measurement results returned from it. Meanwhile, the OLED display presents the real-time status of spectral data acquisition and displays the detection results fed back from the host computer. The physical module is illustrated in Figure 2e.

#### 2.2.2. The Design of the Spectral Acquisition Module

The light source of the instrument needs to meet the following conditions: first, the spectral range should be no less than 350 nm to 1050 nm; second, the illuminance of the light source must be sufficient for transmission through the apple; third, the manufacturing cost should be low; and fourth, the light source output should be stable. Currently, there are very few LED products with a wavelength range of 350 nm to 1050 nm, and they are expensive to manufacture. Considering practical requirements, the OSRAM MR16 halogen lamp was chosen between halogen and LED options. The MR16 lamp has dimensions of 505,037 mm, a power rating of 20 W, and operates at 12 V. In conjunction with the portable multi-quality Vis/NIR detection device for red grapes designed by Gao Sheng et al. [40], the vacuum suction ball paired with the PEG-25N vacuum suction cup was selected to address issues such as varying apple sizes, surface irregularities, and ease of measurement. This setup ensures that the spectral collection probe adheres to the apple’s surface and reduces interference from external light during spectral data collection. To ensure the adhesion time of the vacuum suction cup, a 0.5 mm diameter flat-ended stainless steel needle is integrated to slowly balance the internal and external air pressure of the vacuum suction ball.

According to the positional relationship between the light source and the collection probe, near-infrared nondestructive detection can be divided into two collection modes: diffuse reflection and diffuse transmission [41]. In the diffuse reflection mode, the light source and collection probe are on the same side. This mode has been widely used in fruit quality detection and is more commonly applied for static detection. However, because the surface of the apple is relatively smooth, diffuse reflection can produce specular reflection signals. These unwanted specular reflection signals are also collected by the probe and transmitted to the spectrometer, thereby reducing the detection accuracy. Additionally, due to variations in light intensity and vascular bundle distribution, the sugar content on the sunny side of the apple is higher than on the shaded side, with the regions near the skin being higher than the interior, and the regions near the calyx being higher than those near the stem. As a result, apples are biological bodies with uneven internal component distribution. The diffuse reflection collection mode only captures spectral information from the shallow layers of the apple flesh and cannot obtain the overall information of the apple. In contrast, in the diffuse transmission mode, the spectrometer and light source are positioned on opposite sides of the apple, allowing the spectrometer to capture spectral signals that pass through the sample. Guo et al. [42] used various preprocessing methods to compare the impact of the two collection modes on detection accuracy. The experimental results showed that the diffuse transmission mode significantly outperformed the diffuse reflection mode. Xu et al. [43] employed an LDA discriminant model to predict and found that the highest accuracy for apple placement posture was achieved when the apple was irradiated along the equatorial plane, with the stem facing upwards. Based on the above considerations, the instrument uses diffuse transmission for spectral collection, where both the probe and the light source are aligned along the equatorial plane of the apple during on-site detection. The physical diagram of this module is shown in Figure 2a.

#### 2.2.3. Selection of Spectrometers and Other Accessories

The design requirements for the portable apple watercore non-destructive detector stipulate that the spectrometer must be compact without compromising measurement accuracy. In line with these criteria, the AIOX2000-13 spectrometer from Beijing Yuguang Technology was selected. This spectrometer boasts a compact and reliable structure, excellent performance, and low power consumption, capable of capturing spectral information across the 350–1050 nm wavelength range. To ensure stable operation of all device components, a silent micro 2006 turbo cooling fan is installed inside the device enclosure for effective heat dissipation of internal electronic modules. The fan measures 30 × 30 × 10 mm and operates at 5 V. Since the halogen lamp cup is exposed to ambient air, its heat dissipation via natural convection suffices, eliminating the need for additional cooling structures. To meet the power supply demands of the halogen lamp and other electronic components, the power system employs high-capacity rechargeable 18,650 lithium-ion batteries with a capacity of 8800 mAh, providing both 12 V and 5 V DC outputs. Outdoor field tests demonstrate that the battery supports approximately five hours of continuous device operation. The battery’s physical dimensions are 148 × 85 × 28 mm, balancing endurance with the device’s compact form factor. During spectral data acquisition, a tactile micro switch button is used for control. To enhance operational convenience, the button is integrated into the light source handle along with the light source itself. A photograph of the light source handle is shown in Figure 2d.

### 2.3. Main Program Design

**Figure 4:**
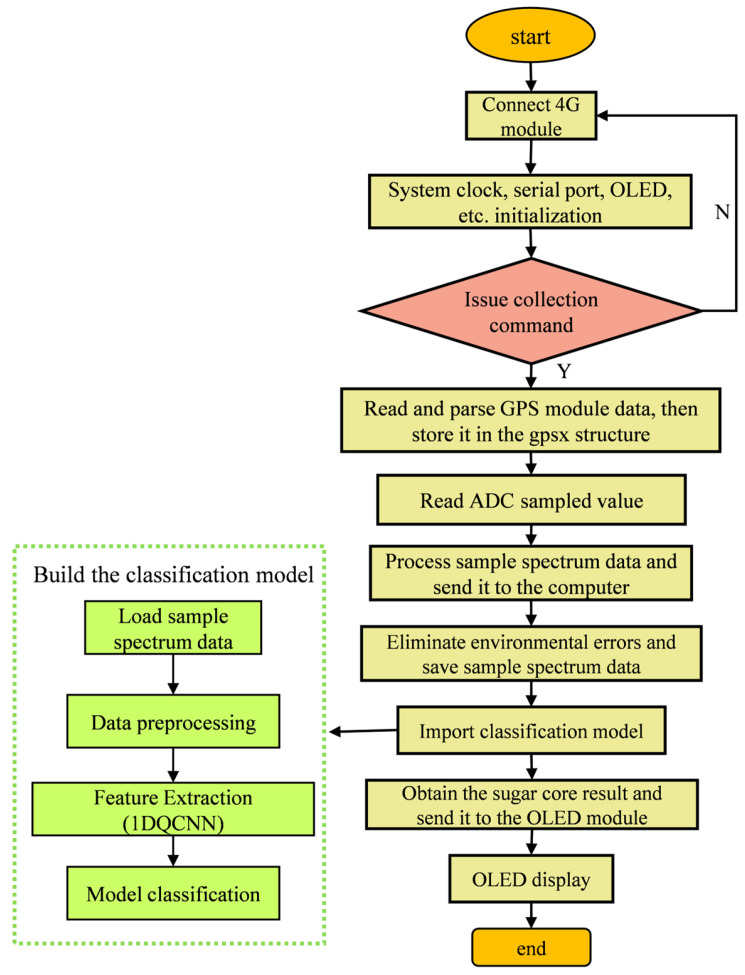
Program flowchart.

The program design of the portable apple watercore non-destructive detector follows this workflow: upon issuance of a collection command, the ARM core processor receives spectral data from the acquisition module and transmits the data to a computer via the 4G module. The computer performs error correction to eliminate ambient light interference on the spectral data and saves the processed information. Subsequently, the refined spectral data is uploaded to the 1DQCNN model for classification. After classification, the detection results are sent back through the 4G module to the display screen for real-time visualization. Upon receiving the next batch of spectral data, the system automatically initiates the subsequent detection cycle. Figure 4 illustrates the program flowchart.

## 3. Development of Watercore Classification Model

### 3.1. Sample Data Collection

#### 3.1.1. Instrument Acquisition Method

During the apple watercore data collection process, the instrument is worn using a waist-mounted configuration: an elastic strap slightly shorter than the device casing secures the instrument vertically at the operator’s abdomen. Once powered on, the display module shows the real-time status of all system components. For formal data acquisition, the operator presses the vacuum bulb to firmly attach the suction cup to the equatorial surface of the apple. One hand stabilizes the vacuum bulb, while the other grips the device handle and aligns the light source with the sample, as shown in Figure 2a. The operator then presses the “Collect” button on the handle (Figure 2d) to start acquiring apple watercore spectral data. After acquisition, pressing the “Send Data” button (Figure 2d) transmits the data to a computer for automatic analysis by the 1DQCNN model, while the 4G communication module simultaneously sends the detection results back to the display module. This completes the full acquisition and classification process for one sample, and subsequent samples follow the same procedure. Overall, from spectral acquisition to result display, each apple takes approximately 5–6 s, including 1 s for spectral acquisition, 2–3 s for data processing and classification, and 1–2 s for result transmission. This rapid response ensures real-time effectiveness on-site, enabling operators to efficiently assess the watercore levels of apples in the orchard. To ensure stable spectral measurements, the spectrometer should be calibrated before experiments using a standard white reference and a dark reference, with periodic recalibration recommended during long-term use. If the measurement environment changes significantly or the fruit variety exhibits abnormal spectral characteristics, the 1DQCNN model may require retraining or fine-tuning. Additionally, temperature, humidity, and surface contaminants on the fruit can affect spectral measurements; therefore, measurements should be conducted under suitable environmental conditions, and the fruit surface should be lightly cleaned to achieve optimal measurement performance.

#### 3.1.2. Data Collection

The test samples in this study consisted of Red Fuji apples from the Ninglang area of Lijiang, Yunnan. According to Peng et al. [44], the optimal harvest period for Fuji apples varies by region but generally falls between September and October. Based on the local farmers’ harvest schedule, the experiment was conducted from 12 October to 5 November 2024, lasting a total of 25 days. During this period, the portable detection device was used daily to collect spectral data from 40 apple samples. Apples were collected from the corresponding orchard areas according to the farmers’ harvest progress. To minimize experimental errors, each sample’s spectral data were collected three times, and the average was taken as the final spectral representation for that apple. In total, 1000 Red Fuji apple samples were collected from the Ninglang orchard. To enhance the representativeness of the dataset and the robustness of the model, samples were stratified according to early-, mid-, and late-ripening stages and measured under different environmental conditions (ambient temperature 5–18 °C; including both sunny and cloudy days). This sampling strategy effectively increased the diversity of the spectral data and improved the model’s generalization ability for practical orchard applications. Future validation on apples from different regions and varieties will be necessary to further confirm the model’s robustness.

**Figure 5:**
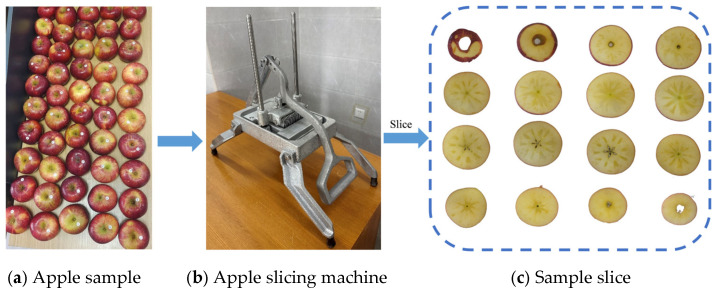
Slices of apple samples.

After spectral acquisition, to further obtain image information of the watercore regions, apples were sliced along the equatorial direction using a custom-made cutting tool, as shown in Figure 5b. This tool ensures that apples are evenly sliced into 4.5 mm-thick sections. Each slice was then visually inspected to determine the presence of watercore areas, and images were captured using a Sony NEX-5T digital camera (Sony (China) Co., Ltd., Guangzhou, China), which has approximately 16.1 million effective pixels, sufficient for high-quality image recording. As shown in Figure 5c, watercore regions appear slightly darker and translucent in the apple slice images. These collected images not only provide a visual representation of the watercore structure but also serve as accurate data for subsequent three-dimensional reconstruction, enabling quantitative analysis of watercore levels.

### 3.2. New Method for Quantifying Apple Watercore Levels

Most current approaches for quantifying apple watercore rely on the area ratio or distribution range of the Watercore region in a single cross-section, which does not adequately capture the spatial distribution characteristics of Watercore throughout the entire fruit. To overcome this limitation, our research team, led by Zhi-Peng Yin [45], proposed a novel method for assessing apple watercore levels based on the RIFE (Real-Time Intermediate Flow Estimation for Video Frame Interpolation) algorithm [46]. RIFE, originally developed by Zhewei Hang et al., is a deep learning-based video frame interpolation technique that employs the Intermediate Flow Network (IFNet) architecture to estimate bidirectional optical flow between two frames in real time and generate high-quality intermediate frames by integrating temporal weights. This algorithm has shown outstanding performance in video enhancement and image generation applications.

**Figure 6:**
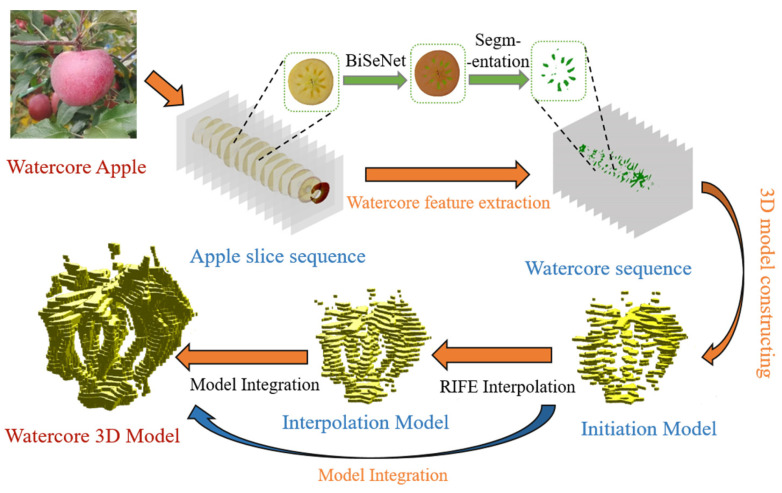
Workflow for Establishing the 3D Model of Apple Watercore.

In this study, the visible/near-infrared (Vis/NIR) transmission spectral data of apples were first acquired, after which the fruits were sliced into 4.5 mm-thick sections using a custom cutting tool, and images were captured for each slice. The BiSeNet network [47] was then applied to perform semantic segmentation on the slice images, extracting features corresponding to the watercore regions. These features were spatially stacked according to the original slice sequence and combined with the slice thickness information to reconstruct an initial three-dimensional model of the apple watercore. To improve the continuity and completeness of the model, the RIFE interpolation algorithm was employed to generate intermediate images between adjacent slices, filling gaps in the original reconstruction and enhancing the coherence of the watercore’s 3D structure. Finally, the initial and interpolated models were fused to produce a complete three-dimensional reconstruction of the apple watercore. By comparing this 3D model with the overall apple volume, the watercore proportion was determined, enabling accurate quantification of the apple watercore degree. The procedure is illustrated in Figure 6. Compared with traditional two-dimensional slice reconstruction methods, this approach improves both the accuracy of watercore region delineation and robustness to image noise and slice misalignment, thereby ensuring reliable three-dimensional volume reconstruction for all tested samples.

### 3.3. Apple Watercore Level Classification Method Based on 1DQCNN and Vis/NIR Spectroscopy Technology

#### Convolutional Neural Network

Traditional convolutional neural networks enhance their representational power by performing dot products between convolutional kernels and the input image (or sequence), followed by the application of nonlinear activation functions. For an input *x*(*t*), the output *f*(*x*) can be mathematically expressed as:

(1) $$\sigma (f(x))=\sigma ((x^{T}w+b)$$

Here, σ(·) denotes the activation function, *w* represents the weight vector, and *b* is the bias term.

CNN extracts features hierarchically from the input, where the lower layers learn general features like contours in an image, while higher layers integrate these lower-level features into abstract high-level representations [48]. Despite the great success of CNNs in image classification, speech recognition, and other domains, their fundamental operation still relies on linear transformations, which might not fully capture more complex nonlinear relationships. To enhance the model’s expressive capability and capture more complex features, some researchers have proposed the quadratic convolutional neural network [49]. The quadratic convolution operation introduces quadratic terms into the traditional convolution operation, allowing it to represent richer features. For an input *x^T^*(*t*), the output f(x) can be mathematically formulated as:

(2) $$\sigma (f(x))=\sigma ((x^{T}w^{r}+b^{r})(x^{T}w^{g}+b^{g})+{\left(x\cdot x\right)}^{T}w^{b}+c)$$

Here, σ(·) denotes a nonlinear activation function; *w^g^*, *w^r^*, *w^b^* are weight vectors, while *b^r^*, *b^g^*, *c* represent bias terms.

Compared with traditional convolution operations, quadratic convolution introduces a quadratic term on top of the standard linear convolution. By convolving with specific quadratic weights, the model can more effectively capture higher-order relationships among input features, thereby enhancing its ability to model nonlinear patterns. Moreover, the squaring operation is essentially equivalent to computing signal autocorrelation, and the autocorrelation property helps the model maintain robust feature extraction even in high-noise environments. Quadratic neurons differ from traditional neurons, which typically perform linear combinations corresponding to piecewise linear functions. In contrast, quadratic neurons can implement piecewise polynomial mappings, thereby enhancing the network’s representational capacity and generalization ability, particularly for handling nonlinear features present in real-world data.

**Figure 7:**
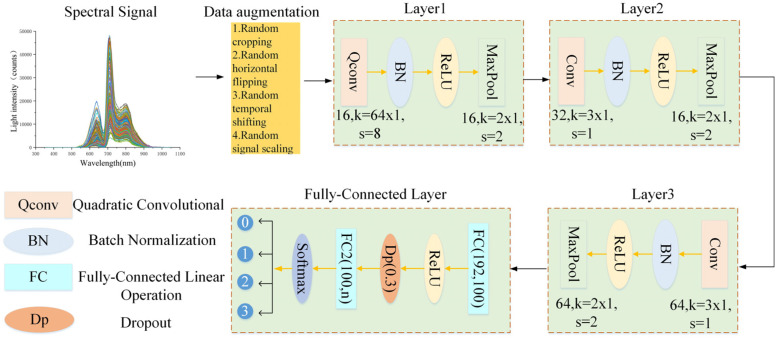
Structure diagram of the 1DQCNN model.

In the 1DQCNN model employed in this study, the first convolution in the conventional 1D-CNN architecture is replaced by a quadratic convolution module, as illustrated in Figure 7. The initial layer uses a large convolution kernel of 64 × 1 with a stride of 8 × 1 to expand the receptive field, reduce data dimensionality, and extract global features, with the number of output channels set to 16. Following the convolution, batch normalization (BN) and ReLU activation functions are applied to improve training stability and enhance the model’s capacity to represent nonlinear patterns. Subsequently, a max pooling layer with a size of 2 × 1 and a stride of 2 × 1 is employed for further dimensionality reduction, reinforcing the extraction of salient.

The second convolution layer keeps both the input and output channels at 16, using a small 3 × 1 convolution kernel and a stride of 1 × 1 to enhance the model’s ability to capture local features. Subsequently, pooling operations are applied again to reduce feature dimensionality and computational overhead. In the third convolution layer, the number of channels is reduced from 16 to 8, with the same convolution kernel size and stride as in the second layer, to balance the model’s parameter count with the retention of feature information. In the fully connected section, the output from the convolutional layers is flattened and compressed from 512 dimensions to 100 dimensions in the first layer. The second layer then outputs an n-dimensional result based on the classification task. A dropout mechanism is introduced to mitigate overfitting and improve the model’s generalization ability. Compared to traditional convolutional network structures, the quadratic convolution module introduced in this paper significantly enhances the model’s ability to extract complex feature relationships from spectral data, leading to higher classification accuracy and stability.

Building on the above analysis, this study proposes a method for classifying apple watercore levels by integrating a one-dimensional quadratic convolutional neural network (1DQCNN) with visible/near-infrared (Vis/NIR) spectroscopy. Initially, the transmission spectral data of apple samples are acquired over a wavelength range of 300–1100 nm, encompassing 701 spectral bands. The BiSeNet network is then applied to perform semantic segmentation of the Watercore regions in the apple slice images. To ensure continuity of the extracted features between slices, the RIFE interpolation algorithm is employed, enabling the construction of a three-dimensional Watercore distribution model. The Watercore degree is subsequently calculated from this model, and the results are used to label the apple samples for training the classification model.

**Figure 8:**
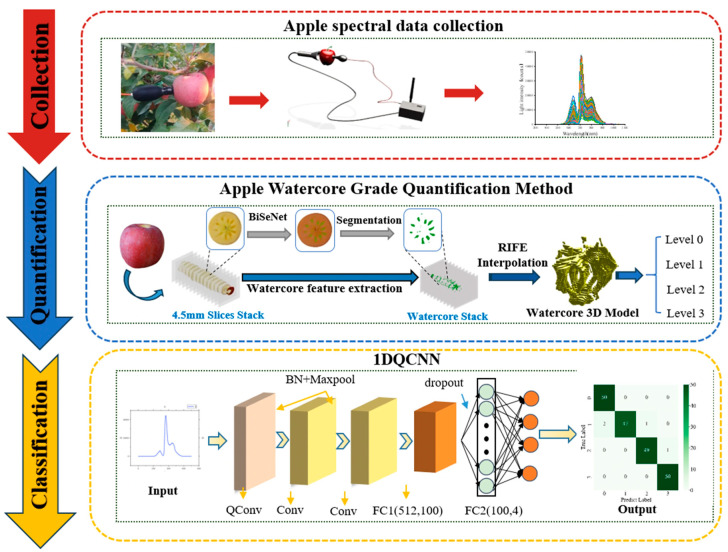
Flowchart.

During the modeling phase, the labeled dataset is partitioned into training and testing sets at a ratio of 3:1. The training set is fed into the 1DQCNN model for feature learning and parameter optimization, after which the trained model weights are saved. The model’s classification performance is then evaluated using the testing set, with classification accuracy recorded. The final weight parameters are preserved for subsequent deployment. The overall structure of this classification process is illustrated in Figure 8.

### 3.4. Results and Discussion

All training and experiments in this study were conducted on a personal computer configured with an Intel i7-13700K processor, an NVIDIA GeForce RTX 4090 24 GB GPU, and running Windows 11. The experimental environment was Python 3.11.4, using a network model built with PyTorch 2.1.0. During model training, the RAdam optimizer was employed with a batch size of 32, an initial learning rate of 0.5, a weight decay of 0.0001, and a total of 500 training epochs to ensure sufficient feature learning while maintaining good generalization performance.

#### 3.4.1. Data Collection Results

**Figure 9:**
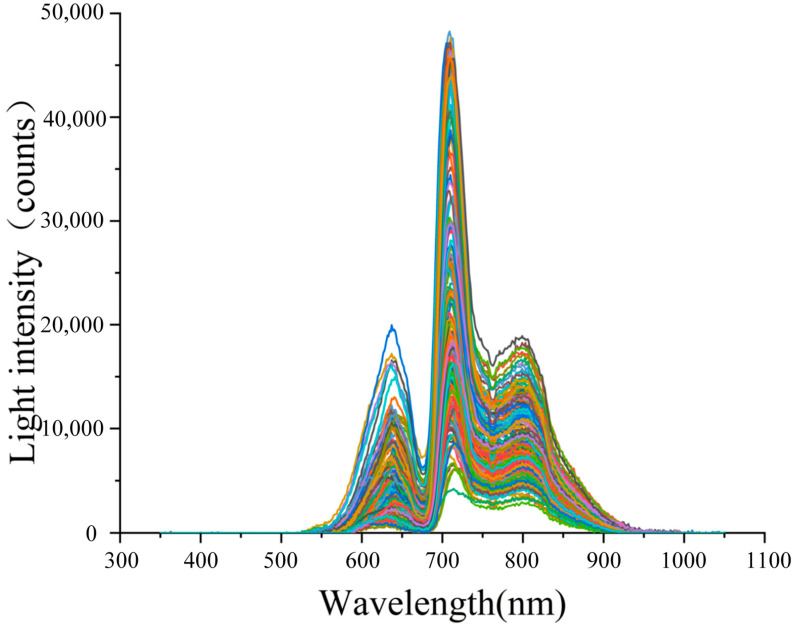
Experimental Data.

The self-made portable device used in this paper collected apple samples. To ensure the accuracy of the model, 200 sample data sets were selected from each grade of the total samples, resulting in a total of 800 sets. The visible/near-infrared spectral data of these samples are shown in Figure 9.

In this study, a total of 800 apple samples were collected, including 600 with watercore and 200 normal apples. Using the previously proposed watercore quantification method, the severity of watercore was calculated for all affected apples, based on which the apples were divided into four levels: normal apples were assigned Level 1; watercore <3% as Level 2; 3–6% as Level 3; and >6% as Level 4. This four-level classification quantitatively reflects the proportion of watercore volume and is consistent with traditional horticultural grading principles, which categorize fruits according to the severity of watercore. Each level included 200 samples.

#### 3.4.2. 1DQCNN Training Results

**Figure 10:**
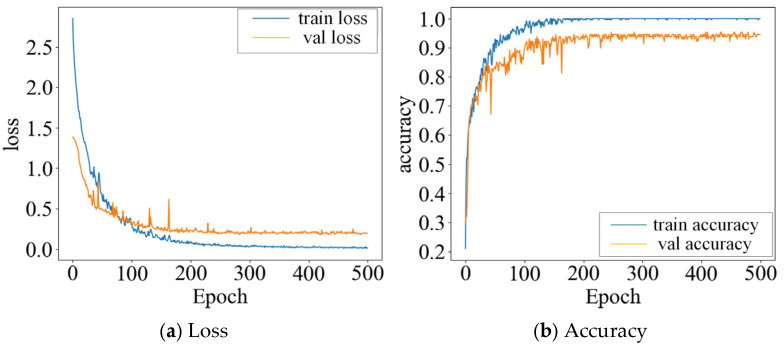
Curve Graph of the test set results.

The quantified watercore data of the apples were used as input, with 800 samples split into training and test sets at a 3:1 ratio: each category included 150 samples for training and 50 samples for testing. Figure 10 shows the accuracy and loss curves of the test set over 500 training epochs. As illustrated in Figure 10a, both training and test losses rapidly decreased with increasing iterations and stabilized after approximately 300 epochs. Figure 10b shows that training and test accuracies increased quickly and plateaued around 180 iterations, indicating good model convergence without obvious overfitting. The final test set accuracy reached 98.05%, demonstrating that the model achieves high classification precision and generalization capability. Compared with methods that convert one-dimensional data into two-dimensional images for classification, the 1DQCNN model provides a more convenient and accurate training process, showing excellent performance in classifying apple watercore levels.

**Figure 11:**
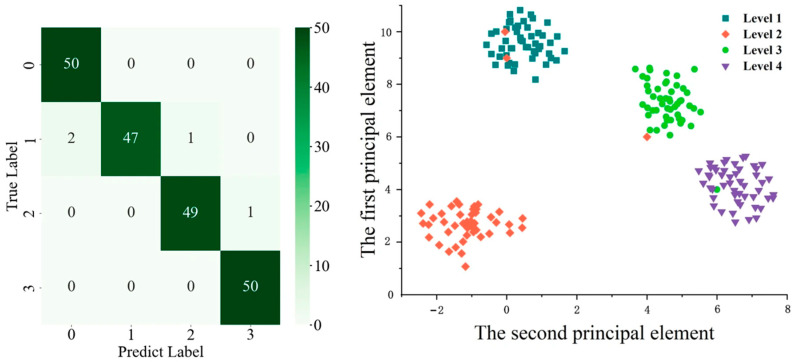
Test set classification results.

The classification results of the testing set for the 1DQCNN model are presented in Figure 11. Figure 11a shows the confusion matrix, where the horizontal and vertical axes correspond to Watercore levels 1 to 4, labeled as 0–3. Figure 11b presents a scatter plot of the classification results, with five colors representing the four Watercore levels. After training the model for 500 epochs using the training set, only 4 out of 200 samples in the testing set were misclassified. Specifically, two apples of Watercore level 2 were classified as level 1, one apple of level 2 was classified as level 3, and one apple of level 3 was classified as level 4. All remaining samples were correctly classified. These results indicate that the 1DQCNN model demonstrates strong classification performance for apples with varying Watercore levels based on visible/near-infrared spectral data.

### 3.5. Comparison of Recognition Effects Between Traditional Methods and 1DCNN

**Figure 12:**
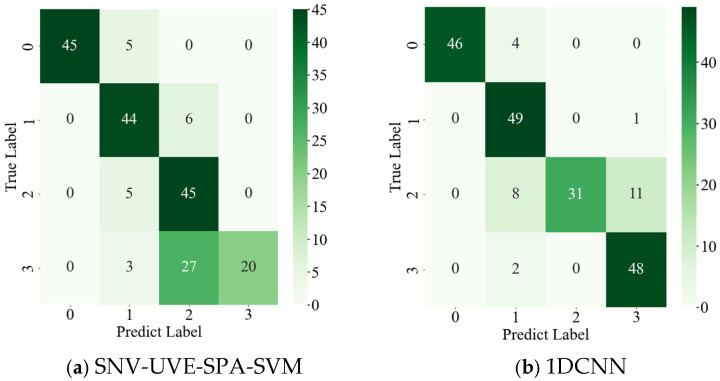
Other methods test the classification results of the test set.

Traditional classification workflows for apple visible/near-infrared (Vis/NIR) spectral data typically involve data preprocessing, feature extraction and selection, followed by pattern recognition with model parameter tuning to construct a classification model. To evaluate the effectiveness of the 1DQCNN model for apple watercore level classification, four preprocessing methods were employed: Max-Min normalization (MMS), Standard Normal Variate (SNV), Multi-Scatter Correction (MSC), and Standardization. Feature extraction was performed using Principal Component Analysis (PCA), Successive Projection Algorithm (SPA), Competitive Adaptive Reweighted Sampling (CARS), and Uninformative Variable Elimination (UVE). Two classifiers—Support Vector Machine (SVM) and Random Forest (RF)—were applied, with data partitioned via the SPXY algorithm. Among these, the SNV-UVE-SPA-SVM combination, with SVM parameters optimized by the Honey Badger Algorithm (HBA), achieved a maximum test set accuracy of 83.81%, as shown in the confusion matrix in Figure 12a.

In contrast, the 1D Convolutional Neural Network (1DCNN) extracts feature patterns directly from raw Vis/NIR spectra, eliminating the need for manual feature design. This approach automatically captures local correlations among key spectral bands, effectively distinguishing spectral differences across Watercore levels. Compared with traditional classification models, the 1DCNN provides advantages such as end-to-end modeling, a simple structure, and efficient training, making it particularly suitable for high-dimensional and continuous spectral data. It achieved a test set accuracy of 89.44%, with the corresponding confusion matrix shown in Figure 12b.

The 1DQCNN model proposed in this study further improves classification performance by incorporating quadratic convolutional layers and optimization strategies. As summarized in Table 1, the 1DQCNN outperforms both traditional methods across all performance metrics, demonstrating superior feature extraction and classification capability for apple watercore spectral signals, and enabling accurate recognition of Watercore levels.

## 4. Experimental Verification

The experimental materials used in this study were Fuji apples from the Lijiang Ninglang production area in Yunnan, with the experiment conducted in a Fuji apple orchard located in Ninglang. The apple sample selection method, instrument testing process, and Watercore level classification model establishment were consistent with previous research. A total of 200 apple samples were tested. To verify the stability of the instrument, each sample was measured five times, and the final Watercore level was determined based on the highest average value of the measurements. The detection results were recorded accordingly. After the testing was completed, all apples were picked, numbered, and transported back to the laboratory. Using a specialized slicing tool, the apples were sliced along the equator. By observing the distribution of Watercore and applying the previously mentioned Watercore level quantification method, the actual Watercore level of each sample was confirmed. Among the samples, 56 apples were classified as level 1, 53 as level 2, 50 as level 3, and 41 as level 4. The detection accuracies for the four levels were 98%, 94.3%, 96%, and 95.1%, respectively, as shown in Table 2. The results indicate that all level 1 apples were correctly identified, with three level 2 apples misclassified, and two misclassifications each for levels 3 and 4. The overall detection accuracy was slightly lower than the model’s performance on the validation set, possibly due to interference from actual environmental factors during data collection. Overall, the portable apple watercore detection instrument demonstrated good accuracy and stability in real-world conditions, with its detection results closely matching the true watercore levels, meeting the requirements for online nondestructive detection applications.

## 5. Summary

This study, addressing the practical need for nondestructive detection of apple watercore, developed a portable online apple watercore grading device based on visible/near-infrared (Vis/NIR) spectroscopy and a one-dimensional quadratic convolutional neural network (1DQCNN). The system, built around the AIOX2000-13 spectrometer and STM32F103VET6 microcontroller and integrated with a 4G wireless communication module, enables efficient acquisition and transmission of spectral data. The instrument is compact, easy to operate, and provides rapid response, fully satisfying the requirements for on-site orchard detection. For model development, Red Fuji apple samples from Ninglang, Lijiang, Yunnan, were collected using a custom spectral acquisition setup. A three-dimensional reconstruction of apple watercore, generated via BiSeNet and RIFE algorithms, was used to quantify and grade Watercore levels, serving as labels for training the 1DQCNN model. Experimental results indicated that the proposed model achieved a classification accuracy of 98.05% on the test set, markedly surpassing traditional spectral modeling methods and standard CNN models, while demonstrating strong noise resistance and generalization capability.

However, this study has certain limitations: First, the establishment of true watercore values relies on image-based indirect quantification, lacking empirical support such as multi-source sensor fusion or manual slicing validation. Second, on-site validation experiments are influenced by environmental illumination and fruit surface variations, so the model’s stability and repeatability need further evaluation under more complex natural conditions. In addition, the cost of the device, system energy consumption, and economic feasibility for mass production require comprehensive consideration.

Future work will focus on four directions: (1) introducing multimodal fusion methods (e.g., combining hyperspectral imaging with near-infrared transmission) to enhance detection accuracy; (2) conducting large-scale field tests across different apple varieties and growing environments to validate model robustness; (3) Further optimize the hardware structure and lightweight algorithm design by developing a more stable mechanical acquisition framework and selecting suitable fabrication materials to enhance the system’s real-time performance, accuracy, and commercial feasibility. (4) Lighting conditions may affect the intensity and uniformity of Vis/NIR signals. Although compensation mechanisms were incorporated into the model, some noise or bias may still remain. Experiments were conducted under relatively stable temperature and humidity conditions, and system evaluation under more diverse environmental conditions was limited. Future work will include testing in different regions and exploring adaptive calibration to enhance robustness and practical applicability.

Overall, the proposed device demonstrates high integration, system stability, and, when combined with a scientific watercore quantification method and advanced deep learning techniques, enables efficient, accurate, nondestructive, and portable grading of apple watercore. It provides technical support for postharvest fruit grading and intelligent quality assessment, showing promising application potential and practical value.

## Figures and Tables

**Table 1:** The table shows the performance of each model in classifying the water core grades.

Classification Model	Evaluation Index		
	F1 Points	Accuracy	Recall Rate
1DQCNN	0.9799	0.9805	0.98
1DCNN	0.8695	0.8944	0.87
SNV-UVE-SPA-SVM	0.7853	0.8381	0.785

**Table 2:** Experimental Results.

Watercore Level	Level 1	Level 2	Level 3	Level 4
Actual Quantity (pcs)	56	53	50	41
Measured Quantity(pcs)	55	50	48	39
Accuracy(%)	0.98	0.943	0.96	0.951

## Data Availability

The data supporting this study can be obtained upon request from the corresponding author. However, due to privacy considerations and the presence of undisclosed intellectual property, these data are not accessible to the public.
